# Structural insights into the specific anti-HIV property of actinohivin: structure of its complex with the α(1–2)mannobiose moiety of gp120

**DOI:** 10.1107/S0907444912040498

**Published:** 2012-11-09

**Authors:** M. Mominul Hoque, Kaoru Suzuki, Masaru Tsunoda, Jiandong Jiang, Fang Zhang, Atsushi Takahashi, Naomi Ohbayashi, Xiaoxue Zhang, Haruo Tanaka, Satoshi Ōmura, Akio Takénaka

**Affiliations:** a Faculty of Pharmacy, Iwaki Meisei University, 5-5-1 Chuodai-Iino, Iwaki, Fukushima 970-8551, Japan; b Department of Biochemistry and Molecular Biology, Rajshahi University, Rajshahi, Bangladesh; c College of Science and Engineering, Iwaki Meisei University, Iwaki, Fukushima 970-8551, Japan; d Graduate School of Science and Engineering, Iwaki Meisei University, 5-5-1 Chuodai-Iino, Iwaki, Fukushima 970-8551, Japan; e KIIM Pharmaceutical Laboratories Inc., Fukushima 970-8551, Japan; f Kitasato Institute for Life Sciences, Kitasato University, Tokyo 108-8641, Japan; g Graduate School of Bioscience and Biotechnology, Tokyo Institute of Technology, Yokohama 226-8501, Japan

**Keywords:** anti-HIV lectins, actinohivin, high-mannose-type glycan

## Abstract

Actinohivin (AH) is an actinomycete lectin with a potent specific anti-HIV activity. In order to clarify the structural evidence for its specific binding to the α(1–2)mannobiose (MB) moiety of the D1 chains of high-mannose-type glycans (HMTGs) attached to HIV-1 gp120, the crystal structure of AH in complex with MB has been determined. The AH molecule is composed of three identical structural modules, each of which has a pocket in which an MB molecule is bound adopting a bracket-shaped conformation. This conformation is stabilized through two weak C—H⋯O hydrogen bonds facilitated by the α(1–2) linkage. The binding features in the three pockets are quite similar to each other, in accordance with the molecular pseudo-threefold symmetry generated from the three tandem repeats in the amino-acid sequence. The shape of the pocket can accept two neighbouring hydroxyl groups of the O^3^ and O^4^ atoms of the equatorial configuration of the second mannose residue. To recognize these atoms through hydrogen bonds, an Asp residue is located at the bottom of each pocket. Tyr and Leu residues seem to block the movement of the MB molecules. Furthermore, the O^1^ atom of the axial configuration of the second mannose residue protrudes from each pocket into an open space surrounded by the conserved hydrophobic residues, suggesting an additional binding site for the third mannose residue of the branched D1 chain of HMTGs. These structural features provide strong evidence indicating that AH is only highly specific for MB and would facilitate the highly specific affinity of AH for any glycoprotein carrying many HMTGs, such as HIV-1 gp120.

## Abstract 2

X-ray analysis of anti-HIV actinohivin in complex with the target α(1-2)mannobiose moiety of high-mannose type glycans attached to HIV-1 gp120 reveals that the three rotamers generated with 120 rotations around the molecular pseudo-rotation axis are packed randomly in the unit cell according to the *P*2_1_2_1_2_1_ symmetry to exhibit an apparent space group *P*2_1_3 as the statistical structure. However, the high-resolution X-ray structure shows the detailed interaction geometry for specific binding.

## 1. Introduction

**Figure 1:**
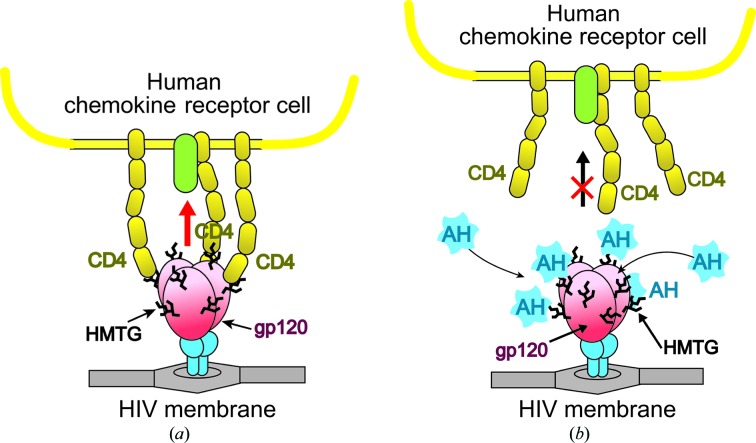
Schematic illustrations of HIV entry into the human cell (*a*) and its blocking by AH (*b*).

**Figure 2:**
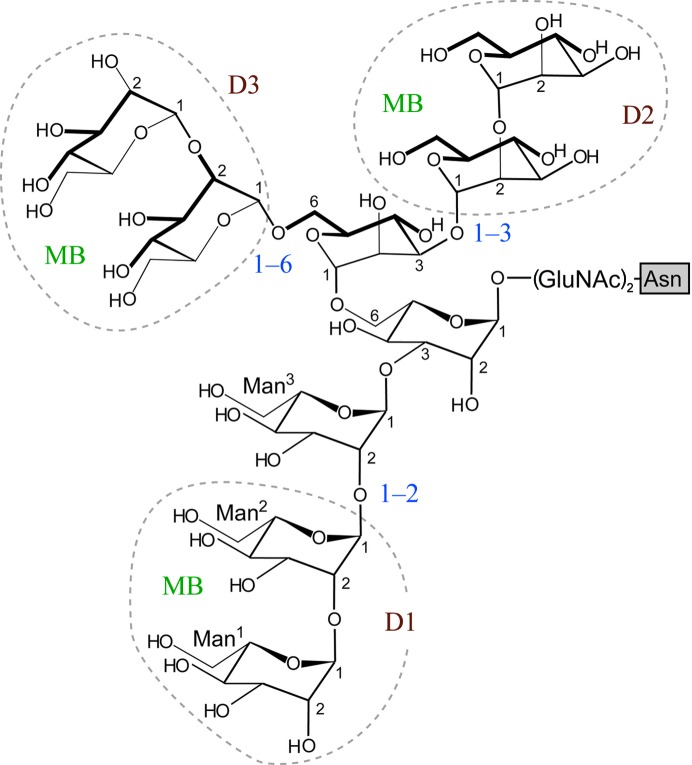
Chemical structure of HMTG. GlcNAc indicates *N*-acetylglucosamine, which is bound to an Asn residue. Here the numbering of mannose residues starts from the end of the branched chain.

HIV/AIDS is a major health concern, a global pandemic which remains a relatively uncontrolled infectious disease. Currently, over 20 kinds of inhibitors targeting HIV enzymes (*e.g.* reverse transcriptase, integrase and protease) are used as medicines to disturb the HIV life cycle after HIV entry into cells (Jegede *et al.*, 2008 ▶). These antiretroviral drugs have recently been evaluated further for their dual effects (Cohen, 2011 ▶) as treatments for and in the prevention of HIV infection (Sigal *et al.*, 2011 ▶; Cohen *et al.*, 2011 ▶). In addition, some proteins which are able to bind the surface glycoprotein of HIV are now expected to prevent HIV entry into cells (Balzarini, 2007 ▶), as shown in Fig. 1 ▶. This effect (entry inhibition) is also applicable to help suppress the spread of infection. Structurally, trimeric gp120 protruding from the HIV surface binds to human CD4^+^ to initiate entry and then to a chemokine receptor: CCR5 or CXCR4 (Berger *et al.*, 1999 ▶). Each gp120 is highly glycosylated (Leonard *et al.*, 1990 ▶) to cover the surface with high-mannose-type glycans (HMTGs; see Fig. 2 ▶). Several carbohydrate-binding proteins (lectins) have been isolated and characterized as candidates for suppressing gp120 binding to susceptible cells. Among them, cyanovirin-N (CV-N) has already been intensively investigated to determine its structural properties, carbo­hydrate-binding potential and antiviral activity (Bewley & Otero-Quintero, 2001 ▶; Bewley *et al.*, 2002 ▶; Fromme *et al.*, 2008 ▶; Tsai *et al.*, 2004 ▶). Another lectin, griffithsin (GRFT), isolated from a red alga (*Griffithsia* sp.), also exhibits a high binding affinity for HMTG of gp120 (Ziółkowska *et al.*, 2007 ▶; Moulaei *et al.*, 2010 ▶). These are strong candidates for development as microbicides to prevent HIV transmission.

**Figure 3:**
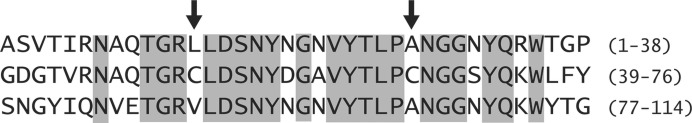
The primary structure of AH. The conserved residues are shaded. The numbering system is shown on the right The arrows indicate two residues which are crucially different between the three repeated sequences.

Similarly, we have independently discovered a new lectin, actinohivin (hereafter designated AH), from an actinomycete, *Longispora albida* K97-0003^T^ (Matsumoto *et al.*, 2003 ▶), which possesses a potent specific anti-HIV activity (Chiba *et al.*, 2001 ▶). This lectin inhibits the entry of various HIV-1 and HIV-­2 strains into susceptible cells, as well as T-­cell-tropic and macrophage-tropic syncytium formation, through binding to HMTGs of the HIV envelope glycoprotein gp120 (Chiba *et al.*, 2001 ▶, 2004 ▶). Previous research (Tanaka *et al.*, 2009 ▶; Takahashi *et al.*, 2010 ▶, 2011 ▶) suggested that the small lectin AH composed of 114 residues (Fig. 3 ▶) binds to three HMTGs at the D1 chain α(1–2)mannobiose (hereafter referred to as MB) moiety of the three branched chains of HMTG (Fig. 2 ▶). AH is thus expected to be developed as another candidate for a useful antiretroviral drug. X-ray analysis of the apo form of AH identified three binding pockets formed tandemly in the single peptide. This structural situation is quite different from those found in CV-N (Botos *et al.*, 2002 ▶) and GRFT (Ziółkowska *et al.*, 2007 ▶), which bind to one HMTG; in particular, GRFT has three binding sites to which the three chains are separately bound (Moulaei *et al.*, 2010 ▶). Therefore, detailed structural knowledge of the binding of AH to MB is essential to modify AH as a useful drug. In the present study, we have successfully determined the crystal structure of AH in complex with MB.

## 2. Materials and methods

### 2.1. Crystallization

AH was purified from a cultured broth of *L. albida* K97-0003^T^ as described previously (Tanaka *et al.*, 2009 ▶). MB was purchased from Sigma Chemical Co. (St Louis, Missouri, USA). Crystallization conditions of AH in complex with MB were surveyed by the hanging-drop vapour-diffusion method at 298 K using crystallization kits from Hampton Research (California, USA) and Emerald BioSystems (Washington, USA). Protein droplets prepared by mixing 2 µl 10 mg ml^−1^ AH solution containing 10 mg ml^−1^ MB in pure water and 2 µl reservoir solution were equilibrated against 700 µl reservoir solution. Several conditions under which crystals appeared were further optimized by varying the concentrations of AH, precipitants and salts at different pH values. Crystals suitable for X-ray experiments grew during 20–30 d using a reservoir solution consisting of 20%(*w*/*v*) polyethylene glycol 1000, 0.2 *M* sodium chloride in 0.1 *M* sodium/potassium phosphate buffer pH 6.2.

### 2.2. X-ray data collection and processing

The crystals obtained were transferred into a cryoprotectant solution (a 1:1 mixture of the reservoir solution and 80% glycerol solution) for 30 s and mounted on a CryoLoop (Hampton Research) for flash-cooling. X-ray data were obtained at 100 K using synchrotron radiation of wavelength 1.00 Å on beamline AR-NW12 at the Photon Factory (PF), Tsukuba, Ibaraki, Japan. A crystal which showed higher order reflections with sharp spots was chosen. Diffraction patterns were taken at 1° oscillation steps with 20 s exposure per frame (a total of 180 frames were obtained) using an ADSC Quantum 210 CCD detector (Area Detector Systems Corp., California, USA). The Bragg spots were indexed and their integrated intensities were scaled between the frames and converted to amplitudes using *iMOSFLM* (Battye *et al.*, 2011 ▶) with *SCALA* in *CCP*4 (Winn *et al.*, 2011 ▶). Crystal data and diffraction processing statistics are summarized in Table 1 ▶. The unit-cell dimensions indicate that the most plausible Matthews coefficient (*V*_M_) is 3.54 Å^3^ Da^−1^ with the cell containing four AH molecules.

### 2.3. Structure determination and refinement

**Figure 4:**
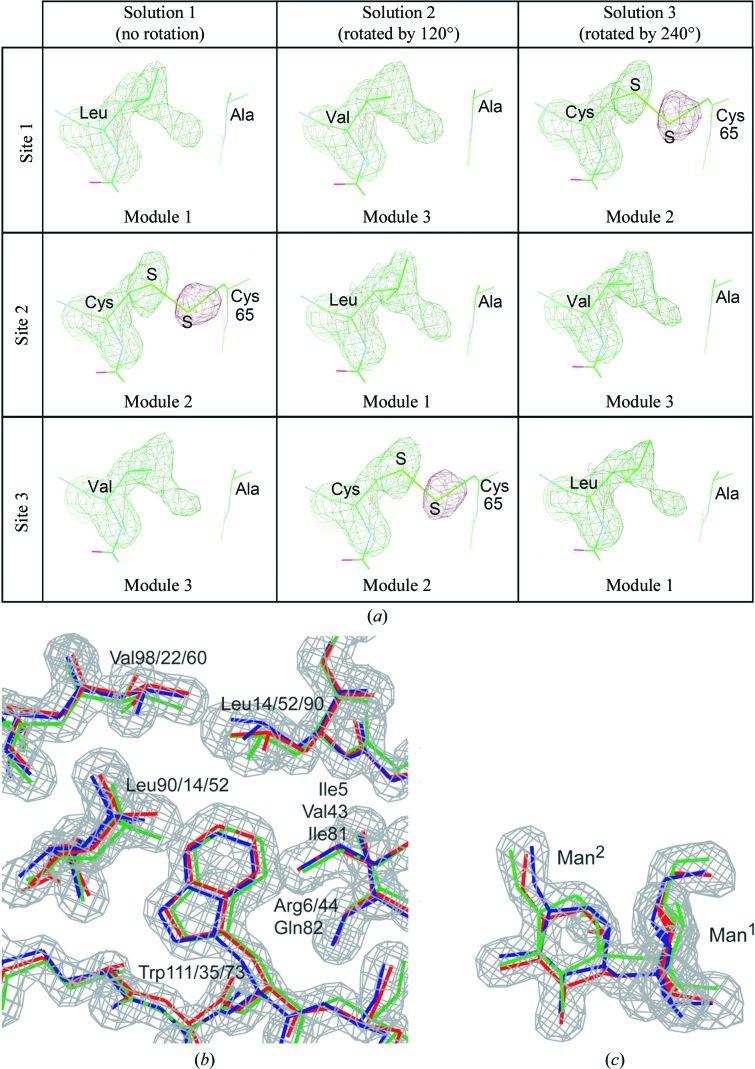
(*a*) *F*_o_ − *F*_c_ OMIT maps at residues 13, 51 and 89 of AH when the three non-disordered crystal structures obtained by the molecular-replacement method were refined separately using the *P*2_1_2_1_2_1_ processed data. From solution 1, the three structural modules of AH are found at three sites in the crystal. In the different solutions, the module rotates between the three sites. Therefore, the amino-acid residues in the same site vary between solutions, *e.g.* Leu13→Val89→Cys51 at site 1. The densities are contoured at the 3σ level. The negative density coloured brown indicates the S atom of Cys65, suggesting that it is not fully occupied. Its residual density appears between the two hydrophobic amino-acid residues in the other modules. Local 2*F*_o_ − *F*_c_ map (contoured at the 1.5σ level) of AH (*b*) and an OMIT map (contoured at the 3.0σ level) of MB (*c*). The three disordered molecules are coloured red, green and blue, respectively.

**Figure 5:**
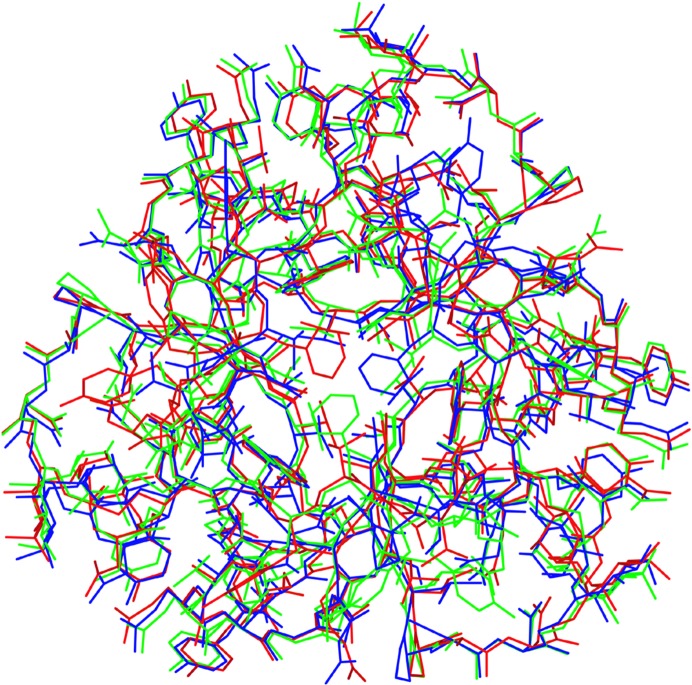
Three disordered AH molecules in the asymmetric unit viewed down the crystallographic threefold axis.

**Figure 6:**
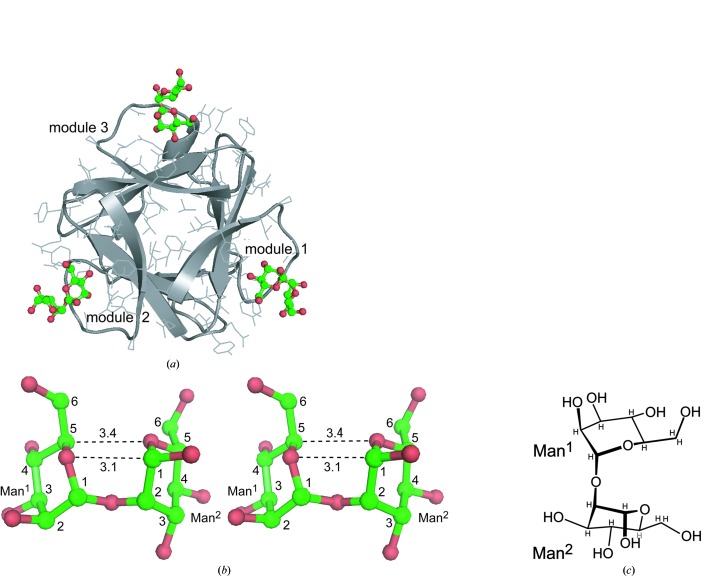
A overall view of AH and MBs bound in the three pockets of the modules (*a*), a stereo diagram (*b*) of MB with bracket-shaped conformation and its chemical structure (*c*). Dashed lines indicate possible C—H⋯O hydrogen bonds with their average distances.

**Figure 7:**
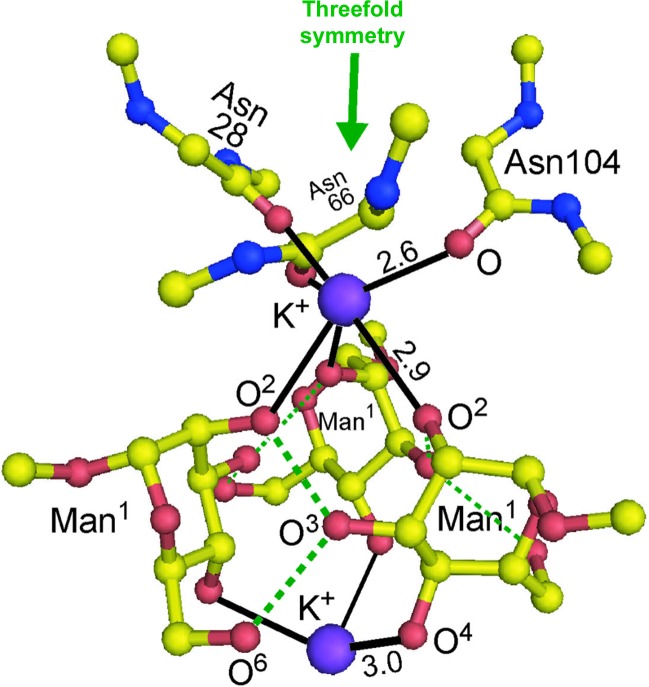
Two potassium ions which cluster the three AH molecules. One of the two potassium ions is surrounded by three Asn carbonyl O atoms and three O^2^ atoms of Man^1^ to form an octahedron. Another potassium ion is bound to three O^4^ atoms of Man^1^, on the opposite side to which several water molecules are disordered. These ligands are related by the crystallographic threefold symmetry. Full black lines show coordination bonds around the two potassium cations (with their distances in Å) and dashed green lines indicate possible hydrogen bonds between the three Man^1^s: O^3^⋯HO^2^ and O^3^H⋯O^6^ in each.

**Figure 8:**
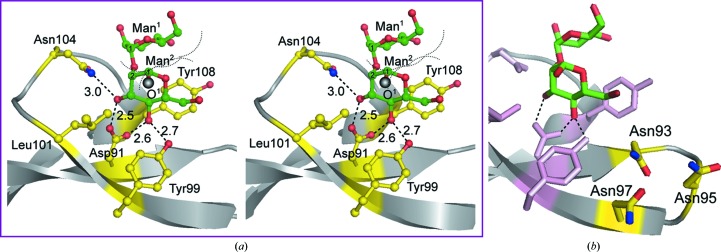
A stereo diagram of MB bound in a pocket of the third module (*a*) and its right side (*b*), where three Asn residues are closely localized. MB adopts a bracket-shaped conformation stabilized by two C—H⋯O hydrogen bonds. The two hydroxyl groups in the equatorial configuration of the second mannose residue are recognized, each by two hydrogen bonds (the average distances are shown in Å). The O^1^ atom is drawn as a grey sphere. Dashed and dotted lines indicate possible hydrogen bonds with their average distances and van der Waals spheres, respectively.

**Figure 9:**
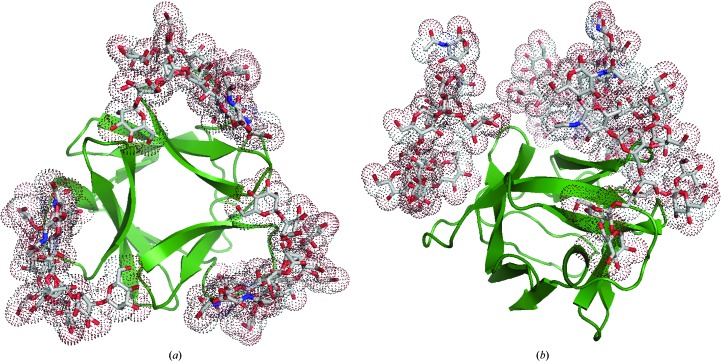
A model of AH bound to three HMTGs drawn in space-filling representation: (*a*) top view, (*b*) side view.

The approximate phase angles of the reflections were estimated by the molecular-replacement technique using *AMoRe* (Navaza, 1994 ▶) from *CCP*4 with the apo form of AH (PDB entry 3a07; Tanaka *et al.*, 2009 ▶) as a phasing probe. The replaced atomic parameters of AH were refined by the restrained maximum-likelihood least-squares technique using *REFMAC*5 (Murshudov *et al.*, 2011 ▶) from *CCP*4. The molecular occupancy was assumed to be one third in the cubic form. The *F*_o_ − *F*_c_ maps showed electron densities assignable to bound MBs, potassium cations and water molecules. After several steps of *REFMAC* refinement with additional atoms, the molecular structures were revised by interpreting OMIT maps at every residue using *Coot* (Emsley & Cowtan, 2004 ▶). The stereochemistry of the protein structures was verified using *PROCHECK* (Laskowski *et al.*, 1993 ▶). Statistical data of the structure determination are summarized in Table 1 ▶. Fig. 4 shows electron-density maps visualized with *DINO* (Philippsen, 2003 ▶) and Fig. 5 shows an overall view of the three disordered AH molecules depicted with *RasMol* (Sayle & Milner-White, 1995 ▶). Figs. 6(*a*), 6(*b*), 7, 8 and 9 were produced with *PyMOL* (DeLano, 2002 ▶). Models of AH with other isomers of mannobiose were constructed using *Coot* and that with HMTG was constructed using *QUANTA* (Accelrys, California, USA).

## 3. Results

### 3.1. Crystal structure

Data processing of the diffraction patterns indicated two possible space groups, *P*2_1_2_1_2_1_ and *P*2_1_3, with the same unit-cell size. They differ in whether or not the unit cell contains threefold axes in the body-diagonal directions. In addition, the calculated *V*_M_ value predicted that the asymmetric unit in *P*2_1_2_1_2_1_ contains a complete AH molecule. A similar example was reported for the crystal of the pseudosymmetric PSPC1–NONO heterodimer (Lee *et al.*, 2011 ▶). *R*_p.i.m._ (Evans, 2006 ▶) would be a reliable index to distinguish between these cases. The calculated values of *R*_merge_ and *R*_p.i.m._ are 0.087 and 0.034, respectively, for *P*2_1_2_1_2_1_ data and 0.089 and 0.029, respectively, for *P*2_1_3 data. The lower *R*_p.i.m._ value for *P*2_1_3 suggests that the latter space group is more plausible. If the cubic space group is chosen, however, the asymmetric unit must contain one third of an AH molecule. This means that an AH molecule is disordered around the crystallographic threefold axis. For an initial examination, structure analyses were attempted using the *P*2_1_2_1_2_1_ data. The application of molecular replacement gave three unique solutions that differed by a ±120° rotation around a body-diagonal axis of the *P*2_1_2_1_2_1_ unit cell. Each solution structure was refined to an *R* factor of 0.213 and an *R*_free_ of 0.235. Fig. 4 ▶(*a*) shows the resultant OMIT maps calculated without the Leu13, Cys51 and Val89 residues (see Fig. 3 ▶), which are related by the molecular pseudo-threefold symmetry within the molecule but differ in amino-acid species between the three modules (Tanaka *et al.*, 2009 ▶). In the second module Cys51 forms a disulfide bond to Cys65, while in the first and the third modules the corresponding residues are Leu13 and Val89, which are exposed to Ala27 and Ala103, respectively. All of the shapes of the densities in Fig. 4 ▶(*a*) are similar to each other in the three sites and in the three solutions, suggesting a mixture of the three amino acids. The negative density coloured brown corresponds to the S atom of Cys65, which was not omitted, but corresponding densities appear in every map of the three solutions. This strongly suggests that Cys65 does not fully occupy one site and that it is distributed to the other sites rotated by ±120°. The remaining densities appear as positive densities between Leu65 and Ala27 and between Val89 and Ala103 in the three solutions.

As described above, intensity distribution statistics and Fourier transformation of high-resolution *P*2_1_2_1_2_1_ data consistently show that an AH molecule is disordered with the crystallographic threefold rotation symmetry in the crystal. Therefore, it is considered that the three AH rotamers generated by ±120° rotation around the molecular pseudo-rotation axis are packed randomly in the unit cell according to the *P*2_1_2_1_2_1_ symmetry and that the whole crystal exhibits an apparent space group *P*2_1_3 as an averaged structure. This type of disorder would be a rare case in which an asymmetric macromolecule crystallizes according to the crystallographic symmetry based on its pseudo-molecular symmetry. For these reasons, the structure determination was performed under the constraints of space group *P*2_1_3 using the *P*2_1_3 data. The refined crystal structure gave a lower *R* factor of 0.145 (*R*_free_ = 0.202). Figs. 4 ▶(*b*) and 4 ▶(*c*) show the final 2*F*_o_ − *F*_c_ maps, in which the three disordered structures are well fitted, as well as those of the bound MB molecules. Whole views of the disordered three AH molecules are shown in Fig. 5 ▶. The atomic coordinates have been deposited in the PDB with accession code 4den. Although the crystal used contained disordered AH molecules, the X-ray diffraction at high resolution and the high symmetry of the AH molecule helped us to successfully reveal the detailed structure of the MB-bound state of AH. A Ramachandran plot of the polypeptide shows that all of the main-chain atoms fall within allowed regions, with 97.3% of the residues in favoured regions and 2.7% of the residues in allowed regions.

### 3.2. Overall structure of AH and MB

In the apo form (Tanaka *et al.*, 2009 ▶), the two AH structures found in the asymmetric unit of the crystal are essentially the same, with an r.m.s.d. of 0.14 Å between C^α^ atoms when they are superimposed onto each other. While the r.m.s.d.s between the MB-bound state and the two apo forms are 0.27 and 0.31 Å, the values for all atoms including side chains are 0.75 and 0.84 Å. These data suggest that the overall conformation is rather rigid, but that the side chains are slightly responsive to target binding. In the apo forms, an additional extension^1^ of two residues at the N-terminus is visible in the electron-density map. In the complex, however, it is difficult to assign these residues even in the final *F*_o_ − *F*_c_ map, despite the protein sample being purified from the same batch. These two N-­terminal residues are invisible, perhaps owing to the packing disorder of the AHs.

Three MB molecules are bound to an AH molecule, as shown in Fig. 6 ▶(*a*). The two mannose residues (Man^1^ and Man^2^) of each MB are close together (see Fig. 6 ▶ *b*), so that two C—H⋯O interactions are made between them. The average C⋯O distances are 3.1 Å between C^1^(Man^2^) and O(Man^1^) and 3.4 Å between C^5^(Man^1^) and O(Man^2^) in the three MBs. Through these weak hydrogen bonds (Desiraju & Steiner, 2001 ▶), the two hexose rings are oriented to have mirror symmetry, apart from the atomic species and the side groups of the rings, as seen in Figs. 6 ▶(*b*) and 6 ▶(*c*), to form a compact conformation as a bracket shape. Similar conformations of MB were also found as a common feature in the lectins CV-N (Botos *et al.*, 2002 ▶) and GRFT (Moulaei *et al.*, 2010 ▶).

Three large peaks were found near the bound MB molecules in the electron-density map, to which potassium ions were assigned because the crystals that were used only appeared when the protein solution contained 0.1 *M* sodium/potassium phosphate buffer. Through the mediation of the two potassium ions, the three AH–MB complexes are associated with each other laterally to form a cluster, as seen in Fig. 7 ▶. The K^1^ atom is bound to the three carbonyl O atoms of Asn28, Asn66 and Asn104 and to the three O^2^ atoms of the Man^1^ residues to form an octahedral coordination. Another K^2^ atom is bound to the three O^4^ atoms of the Man^1^ residues. Therefore, each Man^1^ is bridged between the two K atoms at the O^2^ and O^4^ atoms. These two potassium ions lie on another crystallo­graphic threefold axis crossing the midpoint of the cubic edges, which differs from that passing the centre of every AH molecule. It appears that they facilitate crystallization of AH–MB in space group *P*2_1_3. Indeed, it was difficult to obtain crystals without potassium ions.

## 4. Discussion

The molecular structure of AH is composed of three structural modules associated with a pseudo-threefold symmetry based on tandem repeats in the sequence (Fig. 3 ▶). To detect the conformational rigidity of the three modules, their structures were compared by superimposition of the corresponding C^α^ atoms (Table 2 ▶). In the two apo-form AHs, the r.m.s.d.s between the three modules are as small as 0.25–0.34 Å, suggesting that the structural equivalency of the three modules is highly conserved. This trend is also observed in the complex form of AH, although the r.m.s. deviations are slightly higher. The structural equivalency between the modules may support the high pseudo-symmetry of the molecule. In each module, the three β-strands form a convex slope on the lateral surface and a long loop forms the rim of a shallow pocket. Fig. 8 ▶ depicts an example of the geometry of the third pocket. In every pocket the first mannose residue (Man^1^) of MB is located at the edge of the rim. The second mannose residue (Man^2^) is accommodated by pushing one side (the C^3^, C^4^ and C^5^ atoms) of the hexose ring into the pocket and is trapped by four hydrogen bonds. Asp91 forms double hydrogen bonds to Man^2^, in which one of the two carboxyl O atoms accepts the hydroxyl group attached to the C^3^ atom of Man^2^ (O⋯O distance of 2.5 Å) and another O atom accepts the hydroxyl group at the C^4^ atom of the same Man^2^ (O⋯O distance of 2.7 Å). In addition, the O^3^ atom of Man^2^ accepts the carbamoyl amide N atom of Asn104 (N⋯O distance of 2.9 Å) and the O^4^ atom accepts the hydroxyl group of Tyr99 (O⋯O distance of 2.8 Å). It seems that the Tyr108 side chain wedges between the two Man residues to contact the C^5^ and C^6^ atoms through hydrophobic interaction. The methyl group of Leu101 faces the C^3^ atom of Man^2^ to block its movement, so that the hydroxyl group attached to the C^1^ atom in the axial configuration protrudes into the outside in an upwards direction. These MB-binding features are the same as those in the other modules.

The three MB molecules, each of which adopts a bracket-shaped conformation stabilized through two weak C—H⋯O hydrogen bonds, are separately bound in the three pockets, which are similar to each other in accordance with the molecular pseudo-threefold symmetry generated from the three tandem repeats in the amino-acid sequence. The shape of the pocket for Man binding can accept the two neighbouring hydroxyl groups of the O^3^ and O^4^ atoms of the second Man residue, both of which are in an equatorial configuration. To recognize them, an Asp residue is located at the bottom of the pocket. The Tyr and Leu residues seem to block the movement of the Man^2^ moiety. Furthermore, in each pocket, the O^1^ atom of the Man^2^ residue protrudes into an open space surrounded by conserved hydrophobic residues (Leu101 and Tyr99 in Fig. 8 ▶ *a*). This is a suitable situation for interaction with the third Man residue of D1 (Fig. 2 ▶) when HMTGs are bound, because the hydrophobic regions of Man^3^ could accommodate the binding through hydrophobic interactions. Practically, although AH itself exhibits low solubility in aqueous solution, when amphipathic molecules such as propanol, methyl­pentane-2,4-diol, MB *etc.* are added the solubility increases remarkably. This fact suggests that these exposed hydrophobic residues are involved in interactions with amphipathic MB.

AH exhibits high specificity for the α(1–2)-linked mannobiose moiety of HMTG. Here, it is interesting to examine how the AH pocket discriminates other isomers of mannobiose with different linkages using *in silico* structural modelling. In the case of 1–3 linkages, the two hexose rings cannot adopt a compact bracket-shape conformation: they are extended to have an L-shaped conformation so that it becomes difficult for the O^4^ (equatorial) and O^5^ atoms to enter the pocket. Similarly, other cases with 1–4, 1–6, 2–3, 2–4 and 2–6 linkages also extend the conformation. To maintain a bracket-shaped conformation, the two Man residues are required to be linked together between the hydroxyl groups in the axial configurations. Although such configurations can also possibly occur in 1–1 and 2–2 linkages, it is difficult to stabilize the compact conformation through the two C—H⋯O interactions.

Structural comparisons of AH with other microbicide lectins active towards gp120 provide an understanding of the high AH specificity. CV-N (Botos *et al.*, 2002 ▶) is a dimeric protein that is stabilized by domain swapping between the two subunits and possesses two separate pockets. Each pocket has a W-shaped convex in which the second and third Man residues, linked by an α(1–2) bond, are bound through several hydrogen bonds. Another lectin, GRFT (Moulaei *et al.*, 2010 ▶), that has a different tertiary structure is also a dimeric protein stabilized by N-terminal peptide swapping so that each domain has three binding pockets for one HMTG. These two lectins have neither three tandem repeats in their primary structure nor pseudo-threefold symmetry in their tertiary structure. In contrast, AH adopts a rather rigid structure constructed on a stable scaffold^2^ folded by the molecular pseudo-threefold symmetry, so that it does not change in overall conformation, and binds specifically to the α(1–2)-linked MB end of the D1 chain. In addition, it can bind three HMTGs of gp120 simultaneously as a trivalent microbicide, magnifying its affinity through a cluster effect in which AH binds three HMTGs (1:3 stoichiometry). This unique structure may bring about its highly specific HMTG binding. Furthermore, AH does not exhibit any mitogenic activity (Hoorel­beke *et al.*, 2010 ▶; Matoba *et al.*, 2010 ▶) in contrast to the other lectins, *e.g.* CN-V.

As shown in Fig. 2 ▶, HMTG protrudes from the surface of gp120 and branches into three chains: D1 (with 1–2 and 1–2 linkages), D2 (with 1–2 and 1–3 linkages) and D3 (with 1–2 and 1–6 linkages). Although their ends are commonly the same MB, D1 is preferentially bound to AH and D3 is weakly bound, as described previously (Tanaka *et al.*, 2009 ▶). In addition, AH binds three HMTGs (Tanaka *et al.*, 2009 ▶). The present X-ray structure of AH in complex with three MBs shows that the three pockets are equivalent major binding sites for the MB parts of three D1s. Between the three pockets of AH, it is noted that there are three open spaces (Fig. 8 ▶ *b*) in which Asn/Asp residues are closely localized (Asn17, Asn19 and Asn21 in the first space, Asn55, Asp57 and Ala59 in the second space and Asn93, Asn95 and Asn97 in the third space). These hydrophilic residues could interact with other parts of the HMTGs through hydrogen bonding. By mutation experiments on AH (Takahashi *et al.*, 2010 ▶), it has been confirmed that these residues are responsible for its activity. Based on these structural features, an *in silico* structural model of three-HMTG-bound AH was constructed, as shown in Fig. 9 ▶, in which the ends of three D1 chains are accommodated in the major binding pockets and those of the D3 chains are in contact with the Asn/Asp residues in the open spaces. This binding feature is quite different from that of GRFT, in which the three branches D1, D2 and D3 of HMTG are separately bound in the three pockets. As HIVgp120 is covered by about ten HMTGs, the three equivalent binding pockets of AH can bridge between HMTGs. This multivalent effect could magnify its potent specificity for gp120. Indeed, dimeric AH prepared by linking two AHs with a designed short peptide exhibits further magnification (Takahashi *et al.*, 2011 ▶). From the above-mentioned interpretations, it could be concluded that AH possesses extremely high specificity for MB binding.

Recently, it has been reported that infections involving cell-to-cell spread are markedly less sensitive to antiretroviral drugs and that cell-to-cell spread may provide a barrier to curing HIV infection (Sigal *et al.*, 2011 ▶). Because AH inhibits syncytium formation by Env-expressing HeLa cells and receptor (CD4 and CXCR4/CCR5) expressing cells (Matsumoto *et al.*, 2003 ▶; Chiba *et al.*, 2001 ▶), it is considered to be able to block the cell-to-cell spread of HIV. Therefore, AH would provide a candidate for investigation for the possibility of curing HIV infection. Together with the development of antiretroviral drugs, some types of microbicide lectins, including AH, should be developed as complementary drugs in order to compensate for disadvantages of the various types of drugs, which may induce multiple drug resistance and immunological problems. To overcome these difficulties, we need to continue intensive and extensive research to develop AH as a useful antiretroviral drug, as well as to examine drug combinations which could more effectively suppress the infectious expansion of HIV/AIDS and help to expedite an end to the HIV/AIDS pandemic in the near future.

## Supplementary Material

PDB reference: actinohivin in complex with MB, 4den

## Figures and Tables

**Table 1:** Data-collection and refinement statistics Values in parentheses are for the highest resolution shell.

Data collection	
X-ray source	AR-NW12, PF
Wavelength (Å)	1.00
Resolution (Å)	39.75–1.60 (1.63–1.60)
Observed reflections	167074
Unique reflections	8104 (1160)
Completeness (%)	99.9 (100.0)
*R*_merge_ †	0.089 (0.376)
〈*I*/σ(*I*)〉	18.1 (6.7)
Crystal data	
Space group	*P*2_1_3 (disordered *P*2_1_2_1_2_1_)
Unit-cell parameters (Å)	*a* = *b* = *c* = 56.2
*Z* ‡	1/3
*V*_M_ (Å^3^ Da^−1^)	3.54
Structure refinement	
Resolution (Å)	28.08–1.60 (1.64–1.60)
Reflections used	7647
*R* factor§	0.145 (0.197)
*R*_free_ ¶	0.202 (0.290)
No. of protein atoms	875
No. of ions	3 K^+^
No. of water molecules	71
R.m.s.d.	
Bond lengths (Å)	0.021
Bond angles (°)	2.2
Ramachandran plot (%)	
Most favoured regions	97.3
Additionally allowed regions	2.7

† *R*_merge_ = 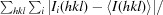 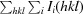, where *I_i_*(*hkl*) is the *i*th measurement of the intensity of reflection *hkl* and 〈*I*(*hkl*)〉 is its mean value.

‡ Number of proteins in the asymmetric unit.

§ *R* factor = 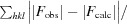 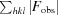, where |*F*_obs_| and |*F*_calc_| are the observed and calculated structure-factor amplitudes, respectively.

¶ *R*_free_ was calculated using a random data set containing 5% of the observations that were not included throughout refinement (Brünger, 1992 ▶).

**Table 2:** R.m.s.d.s (Å) between corresponding C^α^ atoms when they are superimposed on each other Module(*i*,*j*) indicates superimposition between modules *i* and *j*. ApoAH-1 and ApoAH-2 are the two independent AH molecules in the asymmetric unit of the apo-form crystal.

	Module(1,2)	Module(1,3)	Module(2,3)
ApoAH-1	0.30	0.29	0.29
ApoAH-2	0.34	0.25	0.27
AH–MB	0.48	0.39	0.48

## Footnotes

1 It is known that AH is matured by reducing the N-terminal extension gradually during cultivation of the microorganism (Suzuki *et al.*, 2012 ▶).

2 The peptide folding is classified as CBM family 13 (Hazes, 1996 ▶; Gilkes *et al.*, 1991 ▶), which contains many proteins that differ in function but are similar to each other in tertiary structure with a stable scaffold.
